# Usability and Acceptability of Clinical Dashboards in Aged Care: Systematic Review

**DOI:** 10.2196/42274

**Published:** 2023-06-19

**Authors:** Joyce Siette, Laura Dodds, Fariba Sharifi, Amy Nguyen, Melissa Baysari, Karla Seaman, Magdalena Raban, Nasir Wabe, Johanna Westbrook

**Affiliations:** 1 The MARCS Institute for Brain, Behaviour and Development Western Sydney University Westmead Australia; 2 Australian Institute of Health Innovation Macquarie University Macquarie Park Australia; 3 St Vincent's Clinical School University of New South Wales Sydney Australia; 4 Biomedical Informatics and Digital Health School of Medical Sciences, Charles Perkins Centre University of Sydney Sydney Australia

**Keywords:** dashboard, visualization, usability, acceptability, user interface design, health information technology, aged care, clinical, database, development, aged care

## Abstract

**Background:**

The use of clinical dashboards in aged care systems to support performance review and improve outcomes for older adults receiving care is increasing.

**Objective:**

Our aim was to explore evidence from studies of the acceptability and usability of clinical dashboards including their visual features and functionalities in aged care settings.

**Methods:**

A systematic review was conducted using 5 databases (MEDLINE, Embase, PsycINFO, Cochrane Library, and CINAHL) from inception to April 2022. Studies were included in the review if they were conducted in aged care environments (home-based community care, retirement villages, and long-term care) and reported a usability or acceptability evaluation of a clinical dashboard for use in aged care environments, including specific dashboard visual features (eg, a qualitative summary of individual user experience or metrics from a usability scale). Two researchers independently reviewed the articles and extracted the data. Data synthesis was performed via narrative review, and the risk of bias was measured using the Mixed Methods Appraisal Tool.

**Results:**

In total, 14 articles reporting on 12 dashboards were included. The quality of the articles varied. There was considerable heterogeneity in implementation setting (home care 8/14, 57%), dashboard user groups (health professionals 9/14, 64%), and sample size (range 3-292). Dashboard features included a visual representation of information (eg, medical condition prevalence), analytic capability (eg, predictive), and others (eg, stakeholder communication). Dashboard usability was mixed (4 dashboards rated as high), and dashboard acceptability was high for 9 dashboards. Most users considered dashboards to be informative, relevant, and functional, highlighting the use and intention of using this resource in the future. Dashboards that had the presence of one or more of these features (bar charts, radio buttons, checkboxes or other symbols, interactive displays, and reporting capabilities) were found to be highly acceptable.

**Conclusions:**

A comprehensive summary of clinical dashboards used in aged care is provided to inform future dashboard development, testing, and implementation. Further research is required to optimize visualization features, usability, and acceptability of dashboards in aged care.

## Introduction

Health information technologies are increasingly being used in the health care sector, including in aged care, due to their capacity to improve workflow efficiencies and quality of care [1,2]. A technology rapidly gaining momentum in health is electronic clinical dashboards. These typically provide a summary of vital clinical data relating to individual patients to increase users’ understanding of their health care needs and care, display trends in patient-reported clinical outcomes, and support decision-making [3,4]. Limited examples of clinical dashboards currently exist within aged care [5,6].

Aged care has a diverse workforce with varying levels of health and digital literacy. In order to address the needs of older adults (defined as individuals aged 65 years and older) in care, their families, and the workforce, dashboards ideally should be designed to support the perspectives and requirements of all relevant stakeholders in aged care. However, there is limited research on how best to present data to support the interpretation of resident outcomes [7]. Furthermore, while the use of visual information can help reduce information overload and improve understanding of data for users in general [4], it is unclear how different types of visual displays used in dashboards may affect comprehension and decision-making for aged care users.

It has been shown that the way in which information is presented (eg, icon displays vs tables, pie charts, and bar graphs) can impact the accuracy of decisions taken by health professionals [4], but limited work has examined whether interpretation of the visual information is dependent upon the expertise, knowledge, and experience of various dashboard users. Aged care organizations are being encouraged to adopt dashboards to improve the quality of care and resident safety [8]; however, dashboards can be used to communicate information to different users, including patients, clinicians, or others.

The aim of this review was to thus identify the visual features of clinical dashboards that are usable and acceptable to the varied number of users in aged care settings in order to help guide future development, design, and implementation of dashboards in aged care.

## Methods

### Search Strategy

Adhering to recommended procedures for systematic reviews (ie, PRISMA [Preferred Reporting Items for Systematic Reviews and Meta-Analyses] guidelines) [9], we conducted a literature search for peer-reviewed empirical studies until April 27, 2022, using a predefined search strategy in the following databases: MEDLINE, Embase, Scopus, PsycINFO, and CINAHL. Primary search terms were dashboard, aged population, aged care, acceptability, and usability; papers were limited to 2000 to April 2022, human subjects, and in English (see search strategy in Table S1 in Multimedia Appendix 1). To increase the comprehensiveness of the search, we scanned the reference lists and cited documents of included peer-reviewed articles (ie, snowballing) to identify any relevant articles missed by the searches.

### Inclusion and Exclusion Criteria

We included peer-reviewed primary studies reporting a usability or acceptability evaluation of a clinical dashboard for use in aged care environments, including home-based community care, retirement villages, and long-term care (Table S2 in Multimedia Appendix 1). All study designs were included.

### Study Selection

**Figure 1:**
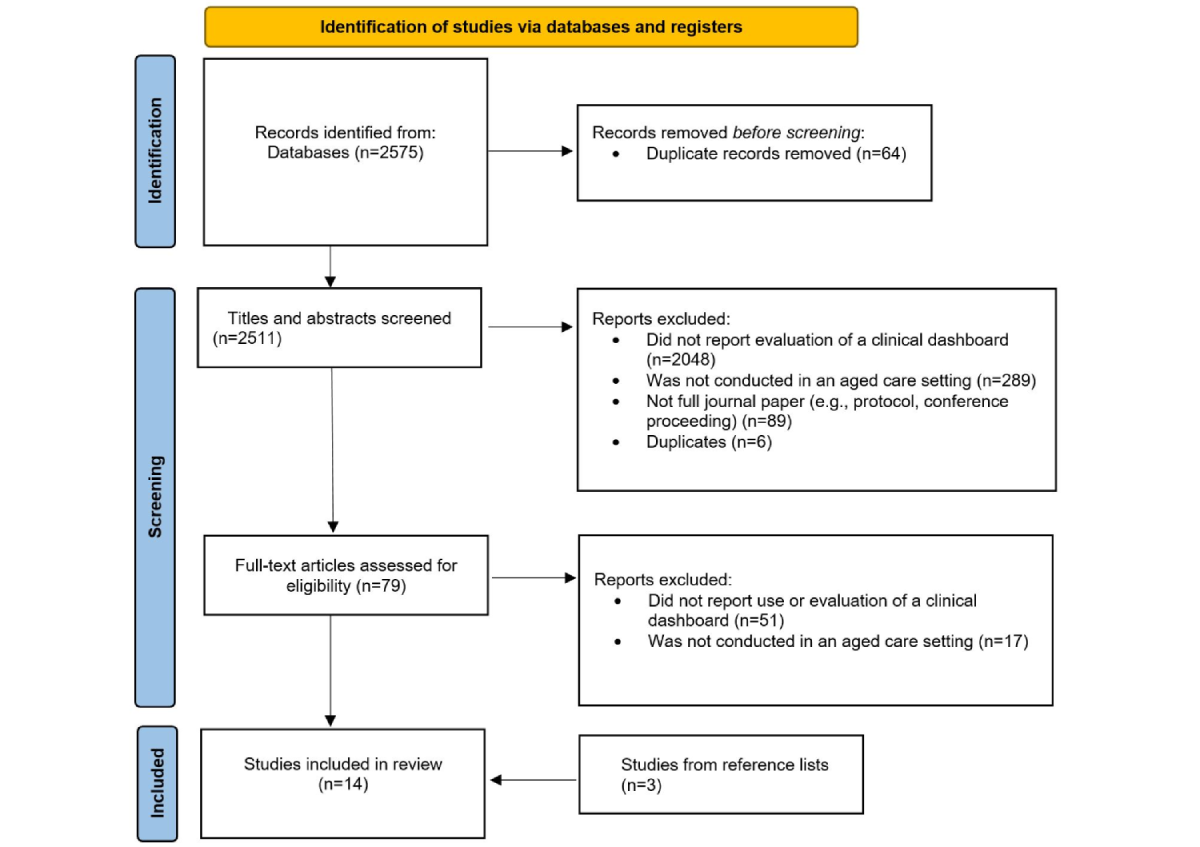
PRISMA (Preferred Reporting Items for Systematic Reviews and Meta-Analyses) diagram.

All potential studies were exported into a reference citation manager and duplicates were removed. The primary author (JS) removed additional duplicates. A random selection of 10% of the abstracts (n=200) was then screened by the 2 authors (JS and FS). An interreviewer agreement was high (100%), with no disagreement on which papers should proceed to full-text screening. FS conducted the remainder of the abstract review. Full-text articles were then obtained for screening by JS and FS, with AN moderating the final list of articles. Please see PRISMA diagram for a detailed summary (Figure 1).

### Data Extraction

#### Overview

Data extraction was completed independently by 2 reviewers (JS and LD) and checked by an additional reviewer (AN). The data extraction tool was piloted to ensure complete documentation of the qualitative and quantitative components of the included studies. Once finalized, data were extracted on study general characteristics (eg, year, country, type of dashboards, participants, and study design), sample characteristics (eg, age and gender), dashboard visual features (eg, charts), acceptability and usability ratings, study findings, and recommendations.

#### Acceptability

Acceptability was defined as the users’ judgement on the appropriateness of the dashboard and its design features, which included sensitivity to their needs as well as usage levels and utility [10]. Adopting the theoretical framework of acceptability [11], perceived user acceptability was explored for the overall dashboard as well as specific design features as described by the study (eg, bar charts). Detailed examples of acceptability scoring are shown in Table 1.

Briefly, acceptability was categorized according to technology acceptability statements in validated technology usability tools or through other in-house developed surveys that were focused on users’ responses to acceptability. For example, statements such as “I found the system unnecessarily complex” in the System Usability Scale [12]; “I think the visual perception of the dashboard is rich” in the Questionnaire for User Interaction Satisfaction [13-15]; and “Using this dashboard would enable me to accomplish tasks more quickly” in the Technology Acceptance Model (TAM) [16] were used to rate acceptability of the dashboards or its features. Acceptability was scored according to the confirmed metrics of these tools and were classified as low, medium, or high, for each scale. For example, with the TAM model, acceptability was defined as low (<50% agreement), medium (50%-70% agreement), and high (>70% agreement) [16].

In-house surveys typically used a 5-point Likert scale of agreement (1=highly disagree to 5=highly agree) to specific statements on the usefulness of the dashboard, its value, and its necessity (eg, Lee and Huebner [17]) and was scored as low (1-2), medium (2-4), and high (4-5) acceptability.

For qualitative articles, general and specific dashboard features that were perceived positively by all stakeholders in a single study were coded as high acceptability, features that included a mix of both positive and negative stakeholder feedback were coded as medium acceptability, and features that were perceived to provide minimal to no added value for stakeholders (eg, low staff engagement [18] or required significant improvements [19]) were categorized as having low acceptability.

**Table 1:** Scoring system for acceptability and usability of articles.

Study design and measurement			Low	Medium	High
**Acceptability**					
	**Quantitative^a^**				
		System Usability Scale [12]	<25	25-35	>35+
		Questionnaire for User Interaction Satisfaction [15]	<5	5-7	>7+
		Technology Acceptance Model [16]	<50% agreement	50%-70% agreement	>70% agreement
		In-house survey on the overall dashboard (eg, “the anticoagulation dashboard is necessary for high-quality home health patient care” [17]) and specific dashboard features (eg, “The graph combining edema status and weight is useful [17])	<50% agreement	50%-70% agreement	70%-100% agreement
	**Qualitative**				
		Participant feedback	Negative appraisals (eg, “The tablet is extra work, and for people with dementia, it’s very important for me to give them extra time.*”* [19])	Containing a mix of both negative and positive comments (eg, “On the right track but not quite there.” [20]; “Whether the system really works remains to be seen. At least it is [better] than nothing.” [21])	Positive appraisal for overall dashboard use (eg, “I find this to be a very helpful tool in a team approach working together with the physician and other team members for the best possible outcomes for our patients” [17]) Positive appraisal for specific dashboard feature (eg, “I have just received one alert, a yellow one, I contacted the older adult the day after...she was happy that it works, and it really works.” [22])
**Usability**					
	**Quantitative**				
		System Usability Scale [12]	<50 (low)	50-70 (medium)	70+ (high)
		Technology Acceptance Model [16]	<50% agreement	50%-70% agreement	>70% agreement
		In-house survey (eg, “The CHF dashboard provides the ease of reviewing necessary patient information at one time.” [17])	<50% agreement	50%-70% agreement	80%-100% agreement
	**Qualitative**				
		Participant feedback	Negative appraisals (eg, “there are no options that we might like to have clicked, that the clients are, for example, chronically or acutely confused.” [19]; “The staff struggled with the challenge of responding to acute events versus detecting trends and patterns of behavioural decline and determining how to integrate such monitoring into their daily schedules” [18])	Mix of appraisals (eg, “We had difficulty logging into the system in the beginning.” [18]; “The system has a learning curve, so training is necessary” but “we can identify fixable usability challenges using scenario based training” [23])	Positive appraisals (eg, “Oh, I love it. I have a sense of being cared for!” [21]; “The electronic form flows nicely. It is set up just like the paper form, is easy to follow and is one less thing on my desk.” [23])

^a^Acceptability subscores of the quantitative scales were used to compute the overall acceptability of the dashboards.

#### Usability

Usability was defined as the extent to which the dashboard could be used by the specified users to achieve their goals effectively, efficiently, and with satisfaction [24,25]. Usability was also rated for overall dashboard use and specific dashboard features using previously described methods focused on usability items in the tools (eg, System Usability Scale, Questionnaire for User Interaction Satisfaction, and TAM) for assessing low, medium, and high usability (eg, Dowding et al [26], Lanzarone et al [27]). These items typically focused on the dashboard’s effectiveness (ie, can stakeholders achieve their goals) and efficiency (ie, amount of effort and resources required to achieve their goal) metrics. For further information, refer to our scoring system described in Table 1.

### Data Synthesis

#### Qualitative Data

For qualitative studies, acceptability and usability were synthesized using a thematic analysis [28] where main themes regarding the acceptability or usability of the dashboard (including its individual visual features) were first identified independently by JS and LD. Any discrepancies that arose were solved through discussion with the third member of the review team (AN). Themes were reviewed and amended by the review team and were subsequently organized into overarching topics for clarity and conciseness. A similar process was also adopted identifying the recommendations to improve acceptability and usability. Where possible, synthesis was made according to different dashboard user types (eg, resident, caregiver, health care professional).

#### Quantitative Data

A narrative synthesis of quantitative articles was used to specify whether clinical dashboards and their features were considered acceptable and usable. Interreviewer disagreement on data extracted was resolved through discussion among the research team. The review team included academics with backgrounds in psychology (JS), aged care (LD and KS), public health (FS and MR), epidemiology (JW, MR, and KS), digital health (JW, AN, MR, and MB), pharmacy (KS, MR, and NW), human factors (MB), and data science (NW). The results were synthesized as a narrative review.

#### Quality Assessment

Study quality was assessed using the Mixed Methods Appraisal Tool (MMAT) [29] by three authors (JS, KS, and MR). This tool allows the appraisal of the methodological quality of 5 categories of studies: qualitative research, randomized controlled trials, nonrandomized studies, quantitative descriptive studies, and mixed methods studies. Each study category has 5 assessment criteria, which are scored as either “yes—criterion met,” “no—criterion not met,” or “unclear/can’t tell whether criterion met” [29]. Mixed methods studies are assessed against the relevant study categories, as well as the mixed methods studies category.

Two reviewers independently scored each study, and disagreements were discussed with a third reviewer to come to a consensus on the rating. An overall quality score was assigned to each study following the method described by MMAT [29]. The score was the overall percent of quality criteria met for an individual study. For multimethod studies, the overall quality score was the score for the lowest-scoring component.

## Results

### Overview

After excluding duplicates, our search strategy identified 2575 potentially relevant articles (Figure 1). After excluding articles that did not meet our inclusion criteria, a total of 14 peer-reviewed articles were included, although 2 articles were reported on the same dashboard [26,30,31] and were described collectively. Articles were most frequently excluded because they did not report an evaluation of a clinical dashboard.

### Study Quality Assessment

The quality of studies ranged from 20% (n=3) to 100% (n=6) on the MMAT checklist (Table S3 in Multimedia Appendix 1) [18-20,22,23,26,27,30-35]. Although more than half of the studies (n=8) received scores greater than 60%, over a third of the studies (n=5) had scores less than 40%, indicating a mix of low, moderate, and excellent quality.

### Characteristics of Studies

Study characteristics are summarized in Table 2. Studies were conducted mostly in the United States (6/12) [17,18,20,23,26,30-32], with 1 study conducted in Australia [33], China [34], Sweden [22], Italy [27], Canada [35], and Europe [19]. The majority of studies adopted a mixed methods design (8/12) [17,18,22,23,26,27,30,35], followed by a quantitative approach (3/12) [20,31,32] and 2 used a qualitative design [19,33]. Studies were carried out mostly in a home care setting (6/12) [17,18,20,22,26,27,30,31], which encompasses domiciliary care, community care, or other social care provided within the home in which the older adult is living or long-term care (6/12) [19,23,32-35], which refers to individuals in residential aged care, nursing homes, or other care facilities that provide permanent accommodation for persons who require consistent and ongoing services to assist with activities of daily living. Studies had varied sample sizes of users (median 32, range 3-292 [22,30]). Most studies described the experiences of health professionals including nurses (9/12) [17,22,23,26,30-35], aged care staff (5/12) [18,19,27,33,35], physicians (3/12) [20,32,35], with 5 studies including a mix of older adults in home or community care, respite care, and long-term care; staff; and health care professionals [18,22,27,32,35].

A summary of the methodological frameworks and theories used to develop or evaluate the dashboards is provided in Table S4 in Multimedia Appendix 1 [16,17,30,32,35-46]. Most dashboards (8/12) used a developmental framework [17,20,22,23,26,27,30,34,35], including feedback intervention theory [47], and most also used an evaluation framework (7/12) [19,22,23,26,27,30,34,35], with the most common being the TAM [16] and the UK’s Medical Research Council complex intervention evaluation framework [48].

**Table 2:** Characteristics of included studies (n=14).

Author (year), country				Study design^a^		Dashboard type		Platform		Software used		Focus^b^		Study setting									System users				
														HC^c^		R^d^		LTC^e^		O^f^		Sample size, n			Age (years), mean (SD)		Sex (female), %
Algilani et al [22] (2016), Sweden				MM		Clinical		ICT^g^ application		In-house		Health status		✓													
																					Older adults: 8			77.6 (—^h^)		60	
																						Nurses:3			—		100
Bail et al [33] (2022), Australia				Qual		Clinical		ICT application		Humanetix		Administrative, health status						✓				Staff: 65			—		—
Bell et al [32] (2020), USA				Quant		Clinical		Web-based		Unclear		Medication and prescribing practices				✓		✓		✓^i^							
																						Older adults: 112			83^j^ (—)		0
																						Physician: 6			—		—
																						Nurse: 1			—		—
Cui et al [34] (2018), China				Quant		Clinical prototype		Mobile app		Unclear		Administrative, health status						✓		✓^k^		Nurses: 18			—		100
Dowding et al [30] (2018), USA				Quant		Clinical prototype		Paper-based		In-house		Administrative, health status		✓								Nurses: 195			49 (11)		89.7
Dowding et al [31] (2018), USA				MM		Clinical prototype		Computer		Morae software (Techsmith)		Health status		✓								Nurses: 292			—		—
Dowding et al [26] (2019), USA				MM		Clinical prototype		Web-based		Morae software (Techsmith)		Health status		✓								Nurses: 32			51^l^ (10)		91
Kramer et al [20] (2016), USA				Quant		Clinical simulation		Computer		In-house		Medication and prescribing practices		✓								Physicians: 19			39.8 (6.1)		57.9
Lanzarone et al [27] (2017), Italy				MM		Administrative		DiamondTouch table		Geodan		Administrative		✓								Staff/other^m^			—		—
Lee and Huebner [17] (2017), USA				MM		Clinical prototype		Computer		MS Excel		Administrative, health status		✓								Nurse: 14			—		—
Mei et al [23] (2013), USA				MM		Clinical		Computer		MS InfoPath, Sharepoint		Adverse events						✓				Nurse: 4			—		—
Papaioannou et al [35] (2010), Canada				MM		Clinical, MEDeINR^n^		Web-based		In-house		Medication and prescribing practices						✓									
																						Older adults: 128			85.9 (8)		75
																						Physician: 4			—		—
																						Nurse: 8			—		—
Shiells et al [19] (2020), Belgium, Czech Republic, and Spain				Qual		EHR^o^		Computer, tablet		Unclear		Administrative, health status						✓				Staff (21)^k^			—		90.5
Wild et al [18] (2021), USA				MM		Clinical, ambient		Web-based		ZigBee		Administrative		✓													
																						Older adults: 95			86.4 (7.4)		80
																						Staff: 25			—		—

^a^Study design (MM: mixed methods; Quant: Quantitative; Qual: Qualitative).

^b^Focus of dashboard (Health status: vital signs, physiological, and functional status, eg, weight, blood pressure; Medication and prescribing practices: medication discrepancies, appropriate prescribing practices; administrative includes care pathways and changes to services/care an older adult is receiving; Falls refers to the incidence of older adult falls).

^c^HC: home or community care. Refers to in-home care, domiciliary care, community care, and social care provided within the home in which the older adult is living compared to care provided in group accommodation, clinics, and nursing homes, and also 3 independent living retirement communities.

^d^R: respite care. Refers to planned or unplanned short-term care for older adults to provide a temporary break for caregivers.

^e^LTC: long-term care. Refers to those in residential aged care, nursing homes, or long-term care facilities who provide permanent accommodation for those who require consistent and ongoing services to assist with activities of daily living.

^f^O: other.

^g^ICT: information and communication technology.

^h^Not available.

^i^Refers to short stay/transitional care and palliative care.

^j^Only at-risk older adults receiving care (n=21) data were reported.

^k^Refers to a community hospital.

^l^Age reported for usability component only.

^m^Including home care planners, experts, and nonexperts of home care providers. Sample size is not provided.

^n^MEDeINR: an electronic decision support system based on a validated algorithm for warfarin dosing.

^o^EHR: electronic health record.

### Dashboard Purpose and Features

An overview of dashboard type and purpose are shown in Table 3. Dashboards were either already established in existing information systems (8/12) [18,19,22,23,27,32,33,35] or were prototypes (4/12) [17,20,26,30,31,34]. Most dashboards were accessed through a computer (5/12) [17,19,20,23,26] or specialized hardware (eg, DiamondTouch table [27]) or a web-based platform (4/12) [18,26,32,35] (Table 2).

The main purpose of dashboards was grouped into four categories: (1) health status (8/12) [17,19,22,26,30,31,33,34], which included monitoring of vital signs, physiological, and functional status such as weight and blood pressure; (2) medication and prescribing practices (3/12) [20,32,35], which referred to medication discrepancies and appropriate prescribing practices; (3) administrative (7/12) [17-19,27,31,33,34], which included exploring and viewing older adult care pathways as well as changes to services or care that the older adult is receiving; and (4) adverse events (1/11) [23], which refers to the specific incidence of falls or other behavior-related events.

Dashboard features are described in Table 3 and were broadly categorized into information, analytic capability, and other functionalities. Most *information* depicted on dashboards included health conditions prevalence and incidence (9/12) [17,18,22,23,26,30-33] and medication use patterns (6/12) [17,18,20,32,33,35], which could be displayed over time (8/12) [17,18,22,26,27,30,32,33]. Other information included geographical location (2/12) [18,27], hospitalization data (2/12) [18,31], and linkage to additional resources of complementary information and guidelines (2/12) [27,32].

*Analytic capability* referred to the dashboard’s ability to display data in a meaningful way (eg, wound record, medical status, and medication administration and use) either through descriptive analysis (12/12) [17-20,22,23,26,27,30-32,34,35], predictive ability (7/12) [17,18,22,26,30,32,35], or prescriptive capability (7/12) [17,18,26,30,32,33,35] (ie, predicting what action should be completed according to available guidelines), which was supported by a visual exploration of the data through charts or other graphical means (6/12) [17,18,20,26,30,31].

*Other functionalities* included interactive forms dedicated to client assessment and service planning (11/12) [17,19,20,22,23,26,27,30,32-35], which included initial assessments, transitions in client care, client-level monitoring (eg, vital signs), as well as the management and coordination of aged care service operations to suit clients’ needs. The ability for stakeholders to communicate and interact was also described (6/12) [17,18,20,23,27,32].

**Table 3:** Summary of dashboard features and functionalities including visual application and analytic capabilities.

Author (year)	Visual representation of information								Analytic capability									Features				
	General					Specific			Descriptive		Predictive^a^		Prescriptive^b^		Visual exploration^c^		Epidemiologic monitoring or surveillance			Client assessment and service planning^d^		Stakeholder communication and interaction^e^
	Prevalence/incidence^f^	Spatial^g^	Resources^h^	Events over time^i^	Medication use patterns		Hospitalization															
Algilani et al [22] (2016)	✓			✓				✓		✓									✓			
Bail et al [33] (2022)	✓			✓				✓				✓							✓			
Bell et al [32] (2020)	✓		✓^j^	✓	✓			✓		✓		✓				✓			✓		✓^k^	
Cui et al [34] (2018)								✓											✓			
Dowding et al [30] (2018)	✓						✓	✓						✓								
Dowding et al [31] (2018)	✓			✓				✓		✓		✓		✓					✓			
Dowding et al [26] (2019)	✓			✓				✓		✓		✓		✓					✓			
Kramer et al [20] (2016)					✓			✓											✓		✓	
Lanzarone et al [27] (2017)		✓	✓	✓				✓						✓					✓		✓^l^	
Lee and Huebner [17] (2017)	✓			✓	✓			✓		✓		✓		✓					✓		✓	
Mei et al [23] (2013)	✓							✓											✓		✓	
Papaioannou et al [35] (2010)					✓			✓		✓		✓							✓			
Shiells et al [19] (2020)								✓											✓			
Wild et al [18] (2021)	✓	✓		✓	✓		✓	✓		✓		✓		✓							✓	

^a^Refers to dashboard/application capability of predicting what could happen (eg, dashboard triggers alerts on older adults with high risk based on risk assessment modeling of older adult health concerns).

^b^Refers to dashboard/application capability of recommending what should be done according to guidelines (eg, decision support).

^c^Refers to any graphical representation of data (eg, charts, graphs, and maps).

^d^Includes initial assessment and transitions in older adult care, monitoring (eg, vital signs), and the management and coordination of aged care service operations to suit older adult needs.

^e^Includes capability of communicating between users of the dashboard and data sharing.

^f^Refers to whether the dashboard/tool provided prevalence or incidence data or indicated the potential to compute these data for reporting purposes.

^g^Refers to visual applications that directly or indirectly provide geographical area or location (eg, of staff and clients).

^h^Refers to whether the dashboard/application provided links to additional physical resources or complementary information, guidelines, and recommendations outside that of the information within the dashboard/application (eg, through links to external websites/files).

^i^Refers to whether the dashboard/application had the capability to display changes in events over time.

^j^Physical resource was a pharmacist to prescribe or deprescribe based on evidence-based guidelines.

^k^Advised the pharmacist of “actionable older adults receiving care” and recommended appropriate prescribing with the provider.

^l^Involved reorganization and allocation of staff and dispatch of emergency vehicles.

### Overall Acceptability and Usability of Dashboards

A summary of the users’ overall perceived acceptability and usability of the dashboards is presented in Table 4. Using the criteria described in the methods, perceived usability was mixed, with 4 studies reporting low [18,19,22,32], 5 medium [20,23,26,27], and 4 high usability [17,30,34,35]. Discrepancies between studies related to whether the dashboard was easy to learn, operate, and navigate, with some stakeholders feeling very confident using the dashboard [34] and others reporting difficulties with dashboard functionalities [17,23,27,33].

In terms of acceptability, most studies reported medium to high acceptance (10/11), with only 1 study revealing low acceptance [19]. While most respondents were willing to use the dashboard in their workplace (eg, 94.4% agreement [34]), uptake was low (eg, across 3 years, more than half of staff members logged in less than once [18]) and initial enthusiasm declined over time (eg, [18]).

There was no distinct pattern of dashboard type (eg, clinical and administrative), platform (eg, ICT application and computer), or focus area (eg, health status, administration, and medication) on reported dashboard usability or acceptability. Older adults tended to report usability as low (3/4 studies) [18,22,32], while other user groups (eg, aged care staff) reported dashboard usability as medium to high (8/9) [17,20,23,26,27,31,33,34]. There were no noticeable differences between users on dashboard acceptability.

**Table 4:** Summary of overall usability and acceptability of dashboard.

Author (year)	Dashboard type	User group (n)	Usability^a^	Key findings	Acceptability^b^	Key findings
Algilani et al [22] (2016)	Clinical	Older adults in-home care (8) Nurses (3)	Low	Interviews*:* Barriers to navigation and access, documentation and monitoring, and subject matter.	High	Interviews*:* Reported acceptability and management of own care.
Bail et al [33] (2022)	Clinical	Staff (65)	Medium	Interviews, focus groups, and survey^c^: Users reported positively on the application across multiple devices, ease of access, scheduling and documentation of information at point-of-care (formatting and structure of alerts), and instantaneity of changes to care plan (rather than waiting hours to weeks). Some users felt that the app interfered with the rhythm of care (eg, repetitive information), lacked training and login for agency staff, resulting in workarounds and missing data, and offering different styles of alerts and flagging (eg, different adverse events and health conditions).	High	Interviews, focus groups and survey^c^: Users reported reduced time spent on information retrieval and documentation; reduced errors by omission and missed documentation; improved staff and resident satisfaction; built consistency working with clinical treatment protocols; assisted management decisions and allocation of resources.
Bell et al [32] (2020)	Clinical	Older adults in respite/long-term/other care (112) Physician (6) Nurse (1)	Low	Survey^c^: Little preference for using dashboard to receive prescribing notifications over traditional methods; user satisfaction, tool integration, and interface intuitiveness.	Medium	Survey^c^: Percentage of time of prescribing recommendations accepted by skilled nursing facilities was adequate (66% uptake).
Cui et al [34] (2018)	Clinical prototype	Nurses (18)	High	Survey: TAMM^d^ found a large proportion of participants who found the dashboard easy to learn, use, and navigate (89%), and were satisfied with the component (100%).	High	Survey: TAMM results highlighting considerable perceived usefulness of the dashboard in improving assessment quality, collecting data, and standardizing information (100% of users).
Dowding et al [30] (2018)	Clinical prototype	Nurses (292)	Medium	Survey^c^: Large percentage of users who were able to use the dashboard immediately (91%) and use icons to switch between data types (96%). Heuristic evaluation and task analysis: Time taken to complete tasks differed (eg, 5.7 minutes for nurses vs 1.4 minutes for expert users).	High	Survey: High SUS^e^ (73.2) and QUIS^f^ (6.1) scores for overall user reactions.
Dowding et al [26] (2019)	Clinical prototype	Nurses (32)	Medium	Survey^c^: >50% of participants had difficulty navigating dashboard and interpreting data in the dashboard due to interoperability.	High	Survey: High SUS (73.2) scores. Interviews: users valued the ability to see trends for vital signs over time.
Kramer et al [20] (2016)	Clinical simulation	Physicians (19)	Medium	Survey: High SUS (86.5) scores, however, reported improvements in accuracy (ie, number of medication reconciliation discrepancies using electronic dashboard vs paper) and amount of time to complete cases (ie, efficiency; reported similar completion time for paper-based process vs electronic dashboard) was mixed.	High	Survey^c^: Majority preferred the electronic module compared to paper-based processes (89.5% of users).
Lanzarone et al [27] (2017)	Administrative	Staff/other (-)	Medium	Survey^c^: Low completion times for task completion, increased distance traveled; however, there was minimal change in nurse allocated to visits (ie, good satisfaction among older adults receiving care) and low numbers of overloaded nurses.	Medium	Survey^c^: Mixed reports on the satisfaction of older adults receiving care, applicability of tool integration, and visualization of the information, with multiple recommendations.
Lee and Huebner [17] (2017)	Clinical prototype	Nurse (14)	High	Interviews: Users provided positive responses regarding the module’s ability to locate laboratory findings quickly, review information easily, and access decision support.	High	Survey^c^: High user ratings of clinical dashboard usefulness and necessity data (100%) particularly for supporting high-quality home health care.
Mei et al [23] (2013)	Clinical	Nurse (4)	High	Survey: High TAM^g^ scores (reported on system usability (eg, time taken to complete, the proportion of participants reporting ease of use) (100%).	High	Survey^c^: High user agreement for improving job performance and accomplishing more work following system implementation.
Papaioannou et al [35] (2010)	Clinical, MEDeINR	Older adults (128) Physician (4) Nursing staff (8)	High	Survey^c^: 100% of users found the platform was easy/very easy to use with improvements in therapeutic range and time in sub/supratherapeutic ranges.	Medium to high	Survey^c^: 75% of users agreed platform decreased workload and 92% felt communication was better. Interviews: feedback found decreased anxiety around prescribing and emphasized improvements for training.
Shiells et al [19] (2020)	EHR^h^	Staff (21)	Low	Interviews: Users reported the absence of core assessment scales in the records, systems being not interoperable, and frustration with organizational support for system access and training.	Low	Interviews: Users reported a low preference for the device (preferring traditional methods of a desktop computer and paper) and its functionality, perceiving it as more work.
Wild et al [18] (2021)	Clinical, ambient	Older adults in-home care (95) Staff (25)	Low	Survey^c^: Low proportion of users who logged into the dashboard (44%). Interviews: users reported technical difficulties and continued unfamiliarity with the system.	Medium	Interviews: Users reported some enthusiasm about interest areas (eg, sleep and medication adherence) and appreciated real-time metrics (eg, sleep duration) being captured.

^a^Usability refers to the extent to which the dashboard could be used by the specified users to achieve their goals effectively and efficiently.

^b^Acceptability refers to the satisfaction with the dashboard and future adoption by specified users.

^c^Survey developed in-house by researchers.

^d^TAMM: Technology Acceptance Model for Mobile.

^e^SUS: System Usability Scale.

^f^QUIS: Questionnaire for User Interaction Satisfaction.

^g^TAM: Technology Acceptance Model.

^h^EHR: electronic health record.

### Dashboard Features

**Figure 2:**
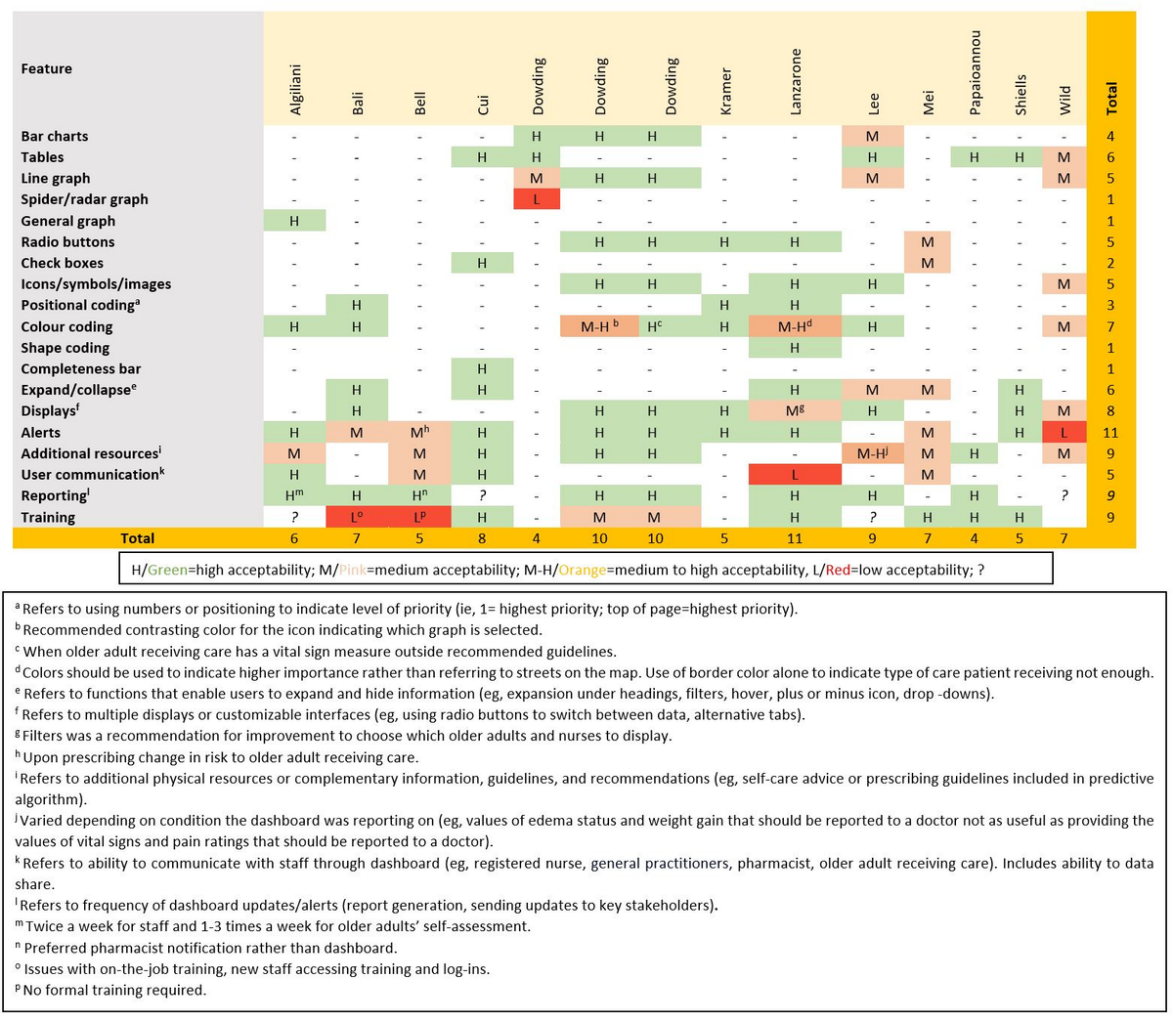
Summary of perceived acceptability to key dashboard features.

An overview of the key dashboard features and their perceived acceptability is provided in Figure 2. The median number of features used in the dashboards was 6 and ranged from 4 [32] to 11 [27]. Displaying an alert (10/13) [17-20,22,23, 26,27,30,32-35] was the most common, followed by customizable displays (8/12) [17-20,26,27,30,33] and the presence of color coding (7/12) [17,18,20,22,26,27,30,33]. One-third of the dashboards used symbols and icons (4/12) [17,18,26,27,30]. Visual graphs such as bar charts (2/12) [17,26,30,31] and line graphs (3/12) [17,18,26,30,31] were less frequently used in the dashboards. Functional aspects, including radio buttons (4/12) [20,23,26,27,30] and checkboxes (2/12) [23,34], were not used frequently.

The ability to update, alert, and generate reports for primary stakeholders was the most frequently used feature and was reported to be highly acceptable across all dashboard types. In general, features with high acceptability were bar charts, tables, icons, symbols, images, and color coding to organize and display information, as well as the use of radio buttons, the ability to expand and collapse information, and multiple displays to facilitate easy customization of the dashboard for different users. A small number of studies also described positional coding, checkboxes, and a completeness bar, which had high acceptability. One study of 195 nurses used a dashboard with spider and radar graphs, and these were reported as too complex [31].

There was only 1 study in-home care exploring older adults’ acceptability for line graphs, icons, and displays, all which were rated as medium. Nurses tended to report communication features (eg, ability to converse with other users in the system) as low to medium [27,32], whereas older adults report it as high [22]. Compared to other user groups, older adults’ acceptability of alert features was variable, ranging from low to high acceptability.

### Problems Identified With Dashboard Acceptability and Usability

Thirteen studies described problems hindering user acceptability and usability of dashboards. The main issues that decreased the overall acceptability and usability of the clinical dashboards included hardware problems, display options, and training. For older adults in home, respite, and long-term care, accessibility of a smart tablet was hindered by locking the tablet, having the incorrect pin code, and forgetting to charge the device [22]. Older adults within each care setting also appreciated a larger text display size and found the 3-step question design difficult when inputting information for a dashboard (eg, yes/no and subsequent questions as they have to recall the previous answer) [22]. For registered nurses, the existing workload prevented daily log-ins despite instructions [18,22]. Similarly, reliance on agency or outsourced workers meant that many staff did not have log-ins and prevented the use of the dashboard [33].

Training on how to use and navigate the dashboard was provided for most dashboard users; however, participant feedback on training ranged from low [32,33] to high satisfaction across studies [19,23,27,34,35]. In some papers, 3 training classroom sessions were sufficient [23], and in others, “on-the-job” training was preferred as an alternative to classroom-based learning [19]. In 1 study, more training was requested by new staff with suggestions for a designated nursing staff member to lead the training session, which could be a recorded session to enable easy dissemination [35].

Suggested areas for improvement across papers mostly related to reducing user workloads, ensuring the security and privacy of resident data, and strengthening decision support and communication features. Ensuring data remain private, particularly data on medication and prescribing patterns, was an emerging area for improvement, with a focus on having data available only to the relevant user [20,32]. Furthermore, inputting reasons for medication use would support nurses’ and clinician’ decision-making on medication administration, identification of discrepancies, and reconciling errors.

Although dashboards could be used to support interactions between different users (eg, staff, providers, and older adults), in 1 study, it was shown that users valued traditional methods of communication, particularly in relation to medication practices (eg, receiving pharmacist notifications separately) rather than logging into the dashboard [32]. This was because users reported spending more time searching for appropriate medication-related information on the dashboard compared to routine practice (ie, predashboard) [20,32] and thus preferred alternative mediums (eg, sourced from electronic notes [32], phone calls [20], and face-to-face conversations [32]) to clarify discrepancies. Suggestions for dashboard functionalities to improve communication and reduce workload included (1) easy-to-navigate workflows [22,27,30]; (2) visual features that allow for better interpretability and usefulness (ie, simple graphs, customizable alerts, and appropriately positioned icons) [19,20,22,31,33]; and (3) timely responses between users to facilitate efficiency and confidence in medication reconciliation and management [20,23,32,35].

## Discussion

### Principal Findings

The aim of this review was to assess current evidence about the acceptability and usability of clinical dashboard features and functionalities in aged care environments. In general, users had high acceptability but mixed opinions on usability, with dashboards focused on administration activities having high acceptability. Dashboards that featured an update, alerts, and reports and those with simple visual elements (eg, bar charts, tables, and symbols) were considered highly acceptable, while those with complex features (eg, spider and radar graphs) had low acceptability.

Clinical dashboards are relatively new in aged care settings, despite these apps being used widely within population health and health services [49]. In our review, dashboards were developed to support a wide range of clinical and administrative purposes and had no distinct pattern of usability and acceptability on dashboard type or platform. Rather, our results suggest that the capabilities of the dashboards and how information is displayed to end users are more likely to influence the acceptability and usability of dashboards.

Previous studies reporting on the usefulness of other dashboard visualization features in health care settings may inform future dashboard design in aged care. For instance, clinicians prefer data tables as they perceive numbers as less “biased” than data that are presented in graphics [50-53]. Although not explored in the studies included in this review, visual aids such as league charts, caterpillar plots, or funnel plots can offer substantial benefits particularly if the purpose of the dashboard includes institutional performance comparisons (eg, comparing several aged care facilities in certain adverse health events). League charts are often desired because of their familiarity and simplicity [50,51]. Caterpillar plots and funnel plots, types of statistical process control techniques, are widely used visual aids for comparing the performance of institutions in certain performance indicator against a benchmark value [54]. Research shows that health care providers prefer caterpillar and funnel plots once they are taught how to read them [52]. A dashboard that includes specific values, as well as organizational comparisons in certain performance indicators may improve service processes and improve delivery of aged care quality [53]. Thus, when designing dashboards, data visualization approaches need to consider the target audience as well as dashboard purpose.

The perceived usefulness and acceptability of dashboards and their features may differ between end users. For instance, in this review, there were differences between older adults and other end users on the perceived usefulness of dashboards, with older adults likely to report usability as low, while other users reported it as medium-high. Such variability in the perceived usefulness of dashboards across end users can be minimized through *customizable design* [55], that is, engaging and considering the need of end users (eg, clients, staff members, and family) in the dashboard development process. A *user-centered design* approach would enable designers to gain an in-depth understanding of end user experiences, expectations, and needs for clinical dashboards, which are critical to addressing usability and acceptability issues and enhancing the likelihood of having an impactful and sustainable dashboard [56,57].

### Implications and Recommendations for Future Dashboard Development

The findings of this study have important implications to guide future dashboard development. Dashboards often focused on 1 aspect of care (eg, clinical or administrative). While clinical outcomes are an important aspect of aged care quality, there is increasing understanding that a holistic resident or client trajectory should be key to understanding quality [58]. Future dashboards thus need to consider and construct an inclusive picture of resident or client needs to support the care continuum from entry in the system.

Our results found that dashboards typically used in-house collected data, with some using real-time reporting of information [18,27,30,35]. As reporting of quality indicators becomes mandatory in aged care sectors in many countries, the use of a dashboard makes it potentially possible to streamline and automate this process. This may relieve aged care staff of the significant time burden in collating and reporting these data [59]. It could also mean that reported data are more accurate as it removes some opportunities for human error and reports in real time.

Given that dashboards present data visually and aim to support users’ decision-making, the use of in-built decision support within a dashboard provides another opportunity for improved quality care. Recommendations in response to information presented in the dashboard could prompt end users to take appropriate actions to improve clinical care [17,26,30,32,35,60]. This review suggests that certain dashboard features are associated with increased usability and acceptability. For example, reduce user workload through customizability and interoperability of the dashboard, provide visual features to support timely interpretation and response, and include links to complementary information to strengthen confidence in clinical decision-making. Extending such decision support to enhance quality care could include alerts for allergies or special care needs, links to published guidelines to make users aware of appropriate care pathways, and medication errors such as duplications and interactions. Implementing evidence-based decision support to inform better care could be seen as highly beneficial within the aged care sector where health literacy levels vary greatly [61,62].

### Limitations

There are several limitations to our review. The exclusion of gray literature, small number of studies fulfilling the inclusion criteria, and poor quality of the included studies are current drawbacks. Furthermore, most of the studies included in the current review did not explore the potential effect of their dashboards on outcomes and care processes (eg, documentation of care processes and better health outcomes). Due to the nature of reporting in each of the study’s findings and the variation in type and size of end user groups, it was not feasible to determine the differences in usability and acceptability between individual groups; thus, our findings are a summary of all respondents. Future research should focus on how the introduction of different types of clinical dashboards could support adherence to quality guidelines and understand dashboard design and usability in terms of mixed versus specific user groups. Identification of areas where dashboards should be most appropriately introduced to target specific initiatives should also be considered (eg, older adults with dementia and home care) to help improve the quality of care. Further work is needed to explore how users understand and interpret dashboard features, their preferences for information presentation, and how the information is used to support care or service planning, decision support, and user behavior.

### Conclusions

Users found dashboards in aged care generally highly acceptable, particularly those with simple visual elements and features such as an update, alerts, and reporting functionalities. This review highlights the variability in the usability of dashboards and identified certain design features of dashboards, which are associated with increased usability and acceptability. Four possible advantageous features and functionalities for future dashboard developments within aged care are emphasized. Specifically, customizability and interoperability to account for different end user preferences; incorporating numerical (tables) and graphical (league and caterpillar charts) presentations of data to facilitate accurate individual assessment and comparison (benchmarking) respectively; integrating changes to client care preferences with real-time clinical outcomes for a holistic representation of the care journey; and building in recommendations and alerts for best practice clinical decision-making to reduce error and support appropriate care pathways. However, further research on the development, testing, and implementation of visualization dashboard solutions to support outcome improvement for older adults is required.

## Appendix

Multimedia Appendix 1 Supplementary material.

## Abbreviations

ICT: information and communication technology
MMAT: Mixed Methods Appraisal Tool
PRISMA: Preferred Reporting Items for Systematic Reviews and Meta-Analyses
TAM: Technology Acceptance Model
