# Supplementary material for: Usability and Acceptability of Clinical Dashboards in Aged Care: Systematic Review
Source: JMIR Aging. 2023 Jun 19;6:e42274. doi: 10.2196/42274 (PMC10334718; doi:10.2196/42274)
Supplement: Multimedia Appendix 1 [file aging_v6i1e42274_app1.docx]

**Supplementary Material**

**Table S1.** Example of a database (OVID Medline) search strategy.

| Constructs | Search terms used |
| --- | --- |
| Dashboard | Exp Dashboard* or information system* or clinical system* or electronic record*) ti.ab |
| AND | |
| Aged care setting | Nursing home* or residential aged care facilit* or long-term care or aged care home ti.ab |
| AND | |
| User perception | Acceptab* or feasib* or usab* or functional* ti.ab |

**Table S2.** Inclusion and exclusion criteria.

|  | Inclusion criteria | Exclusion criteria |
| --- | --- | --- |
| Publication date | 2000–2021 | Before 2000 |
| Language | English | Other than English |
| Document type | Peer reviewed, empirical research papers and literature reviews | Opinion pieces, conference abstracts, theses  Reports describing dashboards without providing any evaluation of results |
| Setting | Aged care including home-based care and long-term care | Inpatient care, tertiary care, primary care |
| Study methods | Qualitative, quantitative, or mixed methods, descriptive studies | Systematic reviews, case studies, opinion pieces |
| Population studied | Adults aged 65 years or over | Adults aged <65 years |
| Relevance to research questions | Relevant to clinical dashboards offered to aged care | Does not adequately discuss clinical dashboards (e.g., other technology types such as apps) |
|  | Sufficient details provided in the document to address research questions | Insufficient details to address research questions |

**Table S3.** Quality appraisal using the MMAT.

| **Study** | **Criteria from the Mixed Methods Appraisal Tool** | | | | | | | | | | | | | | | | | | | | | | | | | **Overall score (%)** |
| --- | --- | --- | --- | --- | --- | --- | --- | --- | --- | --- | --- | --- | --- | --- | --- | --- | --- | --- | --- | --- | --- | --- | --- | --- | --- | --- |
|  | 1.1 | 1.2 | 1.3 | 1.4 | 1.5 | 2.1 | 2.2 | 2.3 | 2.4 | 2.3 | 3.1 | 3.2 | 3.3 | 3.4 | 3.5 | 4.1 | 4.2 | 4.3 | 4.4 | 4.5 | 5.1 | 5.2 | 5.3 | 5.4 | 5.5 |  |
| Algilani (2016) [22] | Y | Y | Y | Y | Y |  |  |  |  |  |  |  |  |  |  | Y | Y | N | N | U | Y | Y | Y | Y | N | 40 |
| Bail (2022) [33] | Y | Y | Y | Y | Y |  |  |  |  |  |  |  |  |  |  |  |  |  |  |  |  |  |  |  |  | 100 |
| Bell (2020) [32] |  |  |  |  |  |  |  |  |  |  |  |  |  |  |  | Y | Y | U | N | N |  |  |  |  |  | 20 |
| Cui (2018) [34] |  |  |  |  |  |  |  |  |  |  |  |  |  |  |  | Y | Y | Y | Y | Y |  |  |  |  |  | 100 |
| Dowding (2018) [30] |  |  |  |  |  |  |  |  |  |  |  |  |  |  |  | Y | Y | Y | Y | Y |  |  |  |  |  | 100 |
| Dowding (2018) [31] | Y | Y | Y | Y | Y |  |  |  |  |  |  |  |  |  |  | Y | Y | Y | Y | Y | Y | Y | Y | Y | Y | 100 |
| Dowding (2019) [26] | Y | Y | Y | Y | Y |  |  |  |  |  |  |  |  |  |  | Y | Y | Y | Y | Y | Y | Y | Y | Y | Y | 100 |
| Kramer (2016) [20] |  |  |  |  |  |  |  |  |  |  |  |  |  |  |  | Y | U | Y | Y | Y |  |  |  |  |  | 80 |
| Lanzarone (2017) [27] | Y | N | N | N | N |  |  |  |  |  |  |  |  |  |  | U | Y | Y | U | U | Y | Y | Y | Y | N | 20 |
| Lee and Heubner (2017) [17] |  |  |  |  |  |  |  |  |  |  |  |  |  |  |  | Y | N | Y | U | Y |  |  |  |  |  | 60 |
| Mei (2013) [23] | Y | Y | N | Y | N |  |  |  |  |  |  |  |  |  |  | Y | Y | Y | Y | N | Y | Y | N | U | N | 40 |
| Papaioannou (2010) [35] | Y | Y | N | N | N |  |  |  |  |  | Y | Y | Y | N | Y | Y | Y | U | Y | Y | N | N | N | Y | N | 20 |
| Shiells (2020) [19] | Y | Y | Y | Y | Y |  |  |  |  |  |  |  |  |  |  |  |  |  |  |  |  |  |  |  |  | 100 |
| Wild (2021) [18] | Y | Y | N | Y | N |  |  |  |  |  |  |  |  |  |  | Y | Y | Y | N | Y | Y | Y | Y | Y | N | 60 |

Y=yes, criterion met; N=no criterion not met; U=unclear or cannot tell whether criterion met. The overall score is the percentage of criteria met. For mixed methods studies where multiple domains were assessed, the overall quality score is the lowest score of the study components [23].

The full MMAT can be found here: <http://mixedmethodsappraisaltoolpublic.pbworks.com/w/file/fetch/127916259/MMAT_2018_criteria-manual_2018-08-01_ENG.pdf>

**Table S4.** Summary of methodological frameworks and models used in developing interactive visualization applications.

| **Author (year)** | **Co-design** | **Dashboard development framework/model/theory** | **Evaluation framework/model/theory** |
| --- | --- | --- | --- |
| Algilani (2016) [22] | Yes | MRC’s complex intervention evaluation framework [52] | MRC’s complex intervention evaluation framework [52] |
| Bail (2022) [33] | Yes | - | Participatory action research design [53] |
| Bell (2020) [32] | Yes | - | - |
| Cui (2018) [34] | Unclear | Integrated-Care-based Precision Care Compass Model [32] | Technology Acceptance Model for Mobile (TAMM) [54] |
| Dowding (2018) [30] | Yes | - | - |
| Dowding (2018) [31] | Yes | Feedback Intervention Theory (FIT) [35] | Tasks, Users, Representations and Functions framework (TURF)  System Usability Scale (SUS) |
| Dowding (2019) [26] | Yes | Feedback Intervention Theory (FIT) [35] | Tasks, Users, Representations and Functions framework (TURF) [55] |
| Kramer (2016) [20] | No | User centered-design principles [56,57] | - |
| Lanzarone (2017) [27] | Yes | V model [58] | V model [58] |
| Lee and Heubner (2017) [17] | Yes | Review and co-design [17,59] | - |
| Mei (2013) [23] | No | Co-design [30] | Technology Acceptance Model (TAM) [16] |
| Papaioannou (2010) [35] | Yes | Knowledge to action framework [60,61] | Knowledge-to-action framework [60,61] |
| Shiells (2020) [19] | No | - | Health Intervention Technology Evaluation Framework [62] |
| Wild (2021) [18] | No | - | - |
